# Contrast sensitivity and higher-order aberrations in Keratoconus subjects

**DOI:** 10.1038/s41598-021-92396-5

**Published:** 2021-06-21

**Authors:** Einat Shneor, David P. Piñero, Ravid Doron

**Affiliations:** 1 Department of Optometry and Vision Science, Hadassah Academic College, Haniviim St. 37, 9101001 Jerusalem, Israel; 2 Department of Optics, Pharmacology and Anatomy, University of Alicante, Alicante, Spain

**Keywords:** Corneal diseases, Perception

## Abstract

This study analyzes the relationship between contrast-sensitivity and higher-order aberrations (HOA) in mild and subclinical-keratoconus in subjects with good visual-acuity (VA). Keratoconus group (including subclinical-keratoconus) and controls underwent autokeratometry, corneal-tomography, autorefraction and HOA measurement. Contrast-sensitivity was tested using a psychophysical two-alternative forced-choice Gabor patches in three blocks (6, 9, 12 cycles/deg). Controls were compared to the keratoconus group and to a keratoconus subgroup with VA of 0.00 LogMar group ("keratoconus-0.00VA"). Spearman correlation tested association between HOA and contrast-sensitivity. Twenty-two keratoconus subjects (38 eyes: 28 keratoconus, 10 subclinical-keratoconus, 20 keratoconus-0.00VA) and 35 controls were included. There was a significant difference between control and keratoconus, and between control and keratoconus-0.00VA, for keratometry, cylinder, thinnest and central corneal thickness (p < 0.001). Controls showed lower HOA and higher contrast-sensitivity for all spatial-frequencies (p < 0.001). Most HOA were negatively correlated with contrast-sensitivity for all spatial-frequencies for keratoconus group and for 9 and 12 cycles/deg for keratoconus-0.00VA. Keratoconus subjects with good VA showed reduction in contrast-sensitivity and increased HOAs compared to controls. HOA and contrast-sensitivity are inversely correlated in subjects with mild keratoconus despite good VA. This suggests that the main mechanism underlying the decreased vision quality in keratoconus is the increase of HOA.

## Introduction

Keratoconus, is a progressive corneal stromal thinning disorder^1^ in which the cornea bulges forward in a cone-shape^2^. In the first stages of the disease, the patient is typically asymptomatic, but as the disease progresses myopia, irregular astigmatism and visual impairment occur^2,3^.

When the disease progress, the cornea becomes less touch-sensitive and clinical signs such as scissoring reflex, Fleischer’s ring, Vogt’s striae, Munson’s sign Rizzuti’s sign, and hydrops can be seen in the cornea^4^. The diagnosis of keratoconus is commonly performed by corneal topography and/or tomography^2,4^ in addition to the typical clinical signs previously mentioned^2,4^. The earliest stage of keratoconus is forme fruste or keratoconus suspect^2^, who have typical topography pattern that is consistent with keratoconus, but without apparent clinical signs^2–4^.

Keratoconus patients perceive a loss of the visual function, which is in most of cases disproportionate to that reflected by clinical measures. For example, some patients report visual reduction despite good best corrected visual acuity^5^. Visual acuity is a measure of the spatial-resolving ability of the visual system under conditions of very high contrast and not at various contrasts which often observed in real life situations. Thus, keratoconus subjects with good corrected visual acuity may report a perceived impairment in vision-related quality of life in areas such as social functioning^6^, emotional well-being^7^ and mental health and dependency^8^.

A better method for evaluation of the visual function in any subject is contrast sensitivity, which is a measure of the threshold contrast for seeing a target^9^. Contrast sensitivity enhances the analysis of visual performance and visual quality provided by visual acuity exam alone, since VA is usually assessing a high-contrast condition^10,11^. Furthermore, it provides important information in the evaluation of functional visual impairment^12^. There are several types of contrast sensitivity tests that usually consist of sinusoidal^13^ or letters target^14–16^ and can be manipulated by frequency, contrast and orientation. Research on contrast sensitivity changes in keratoconus is limited. Some authors have confirmed that contrast sensitivity is reduced in keratoconus subjects compared to normal controls^14,17,18^. Likewise, contrast sensitivity at medium and high frequencies was shown to be reduced in keratoconus subjects before visual acuity decreases^19^.

Considering that higher-order aberrations (HOA), particularly coma aberration, are significantly increased in keratoconus^20–26^, it can be hypothesized that these optical errors are the main cause of visual impairment in this disease. Thus far, three studies have looked at the correlation between HOA and contrast sensitivity in patients with keratoconus. Indeed, they found that increased HOA has been shown to correlate with a decrease in contrast sensitivity^20,27,28^. However, these studies did not look specifically at keratoconus patients with normal VA. The current study aimed to assess contrast sensitivity using a psychophysical approach^12,29^and to measure HOA using a Hartmann-Shack aberrometer to evaluate the correlation between contrast sensitivity and HOA in keratoconus and keratoconus suspect subjects, and specifically, in a subset of subjects having good visual-acuity (VA). The hypothesis of the current study was that the HOA underly the poor vision quality in keratoconus and keratoconus suspect with normal visual acuity.

## Results

This study included 73 eyes of 57 subjects, with a mean age of 25.2 ± 3.7 (range 17–38 years) and mean visual acuity of 0.04 ± 0.09 logMAR (range 0.00–0.50 LogMAR, Table 1). The Keratoconus group included 38 eyes of 22 subjects (14 women) diagnosed as mild keratoconus (grades 1 and 2) or keratoconus suspect (8 subjects with binocular keratoconus, 7 subjects with keratoconus in one eye and keratoconus suspect in the fellow eye, 5 subjects with monocular keratoconus, one subject with binocular keratoconus suspect and one subject with monocular keratoconus suspect) in the age range of 17–38 (mean age of 25.6 ± 5.0 years) and mean best corrected visual acuity of 0.08 ± 0.12 LogMar.

**Table 1:** Demography, mean visual acuity and refraction for keratoconus group, keratoconus subjects with visual acuity of 0.00 LogMar and for control group.

	Keratoconus group	Control group	Keratoconus with VA 0.00 LogMar	p_mann whitney_ Keratoconus versus controls	p_mann whitney_ Keratoconus with 0.00 LogMar versus controls
N (eyes)	22 (38)	35 (35)	14 (20)	–	–
Age range (years)	17–38	20–32	17–38	–	–
Mean age (years)	25.6 ± 5.0	25.0 ± 2.6	25.2 ± 5.7	U = 363.5, p = 0.72	u = 177.5, p = 0.70
Gender (male:female)	14:8	17:18	9:5	*x*^*2*^_2,36_ = 1.24, p = 0.29^†^	*x*^*2*^_2,36_ = 1.00, p = 0.36^†^
VA range (LogMar)	0.00 to 0.50	0.00 to 0.00	0.00 to 0.00	–	–
VA (LogMar)	0.08 ± 0.12	0.00 ± 0.00	0.00 ± 0.00	u = 350.0, p < 0.001**	–
Sphere (D)	− 2.41 ± 2.95	− 0.68 ± 1.80	− 1.29 ± 2.39	u = 941.0, p = 0.002	u = 445.0, p = 0.10
Cylinder (D)	− 2.11 ± 1.90	− 0.59 ± 0.43	− 1.66 ± 1.33	u = 1174.0, p < 0.001**	u = 580.0, p < 0.001**

*Significant level of < 0.05, **Significant level of < 0.005. ^†^Fisher’s exact test was done to test the gender differences between groups.

VA, visual acuity; D, diopter.

Data from one eye was only used in five keratoconus subjects. In two cases, subjects had binocular keratoconus but they did not perform psychophysics measurements in one eye. In another two cases, keratoconus suspect was present in the fellow eye, but subjects did not perform psychophysics measurements in that eye. In one case, topographic measurement was not reliable enough for a correct classification of one eye.

The control group included 35 eyes of 35 healthy subjects (18 women) in the age range of 20–32 years (mean age of 25.0 ± 2.6 years) and mean best corrected visual acuity of 0.00 ± 0.00 LogMar. There was no difference in age (p = 0.72) or gender (p = 0.29) between groups. However, VA (p < 0.001) was better, and both sphere (p = 0.002) and cylinder (p < 0.001) were smaller in control group (see Table 1).

Table 2 shows corneal parameters and HOA for all groups of subjects. There were significant differences between groups for visual acuity, cylinder, keratometric readings, thinnest corneal thickness, central corneal thickness and for the following ocular HOA: total root mean square (RMS) HOA, trefoil, total coma, tetrafoil, high-order astigmatism, HOA spherical aberration.

**Table 2:** Corneal parameters and ocular higher-order aberrations for keratoconus group, keratoconus subjects with visual acuity of 0.00 LogMar and for control group.

	Keratoconus group	Control group	Keratoconus with VA 0.00 LogMar	P_Mann–Whitney_ Keratoconus versus controls	P_Mann–Whitney_ Keratoconus with 0.00 LogMar versus controls
**Scheimpflug/Placido Disc measurement**					
K_1_ (mm)	7.50 ± 0.43	7.89 ± 0.30	7.57 ± 0.43	u = 1055.0 p < 0.001**	u = 538.0 p < 0.001**
K_2_ (mm)	7.26 ± 0.47	7.75 ± 0.30	7.36 ± 0.47	u = 1114.0 p < 0.001**	u = 568.0 p < 0.001**
K_ave_ (mm)	7.38 ± 0.44	7.82 ± 0.30	7.47 ± 0.75	u = 1100.0 p < 0.001**	u = 560.0 p < 0.001**
TCT (µm)	477.25^†^ ± 35.26	532.47 ± 25.61	484.02 ± 32.35	u = 1167.0 p < 0.001**	u = 614.0 p < 0.001**
CCT (µm)	493.73^†^ ± 31.40	536.52 ± 24.11	496.69 ± 28.78	u = 1112.0 p < 0.001**	u = 595.0 p < 0.001**
**Hartmann-shack ocular high-order aberrations**					
Total RMS HOA (µm)	1.14 ± 1.00	0.20 ± 0.13	0.97 ± 1.04	u = 93.0 p < 0.001**	u = 59.0 p < 0.001**
Trefoil (µm)	0.40 ± 0.32	0.09 ± 0.07	0.38 ± 0.36	u = 146.0 p < 0.001**	u = 97.0 p = 0.001**
Total Coma (µm)	0.95 ± 0.94	0.13 ± 0.11	0.80 ± 0.93	u = 169.0 p < 0.001**	u = 97.0 p = 0.001**
Tetrafoil (µm)	0.12 ± 0.07	0.03 ± 0.02	0.11 ± 0.08	u = 95.0 p < 0.001**	u = 75.0 p < 0.001**
High-order astigmatism (µm)	0.27 ± 0.28	0.04 ± 0.04	0.22 ± 0.29	u = 142.0 p < 0.001**	u = 110.0 p < 0.001**
HOA Spherical aberration (µm)	0.18 ± 0.18	0.05 ± 0.06	0.17 ± 0.23	u = 214.0 p < 0.001**	u = 147.0 p < 0.001**

*Significant level of < 0.05, **significant level of < 0.005. ^†^One subjects did not have good Scheimpflug pictures for 1 keratoconus suspect eye, therefore central corneal thickness and thinnest corneal thickness are average results for 37 eyes (not 38).

VA, visual acuity; K, keratometry; CCT, central corneal thickness; TCT, thinnest corneal thickness; RMS, root mean square; HOA, higher-order aberrations; µm, micrometer.

Since contrast sensitivity is known to be correlated with visual acuity^30,31^, contrast sensitivity was also evaluated in keratoconus subjects having good visual acuity. Thus, contrast sensitivity in subjects in the keratoconus group who had visual acuity of 0.00 LogMar was compared with that measured in the control group in which all subjects had visual acuity of 0.00 LogMar (see Tables 1, 2). This group included 20 eyes of 14 subjects (5 females, 2 subjects with binocular keratoconus, 7 subjects with monocular keratoconus, 3 subjects with monocular keratoconus and keratoconus suspect in the fellow eye, one subject with binocular keratoconus suspect and one subject with monocular keratoconus suspect). Despite identical VA, significant differences were observed between groups for cylinder, keratometric readings, thinnest corneal thickness, central corneal thickness and all ocular HOA.

**Figure 1:**
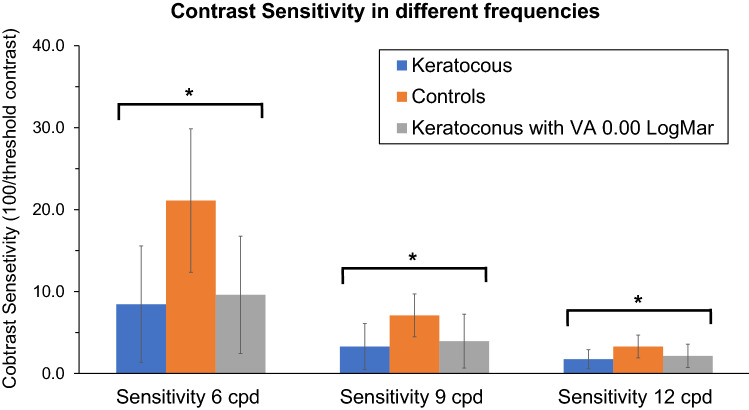
Contrast Sensitivity in different frequencies for keratoconus group, keratoconus subjects with visual acuity of 0.00 LogMar and for control group. Blue bars represent contrast sensitivity for keratoconus subjects, orange bars represent contrast sensitivity for keratoconus subjects with visual acuity of 0.00 LogMar and gray bars represent contrast sensitivity for Control subjects. *Significant level of < 0.005. The figure was generated using Microsoft 365 Excel Version 2103 and the resolution adjusted using Microsoft 365 PowerPoint Version 2103.

Figure 1 illustrates contrast sensitivity results for all groups of subjects. Contrast sensitivity was significantly lower in keratoconus (8.45 ± 7.12, 3.29 ± 2.80, 1.74 ± 1.16 for 6,9 and 12 cycles/deg, respectively) compared to control groups (21.20 ± 8.64, 6.95 ± 2.66, 3.27 ± 1.41 for 6, 9 and 12 cycles/deg; all p < 0.001) and significantly lower in keratoconus with visual acuity of 0.00 LogMar (9.59 ± 7.17, 3.94 ± 3.29, 2.14 ± 1.42 for 6,9 and 12 cycles/deg, respectively) compared to control groups (6 cycles/deg: p < 0.001; 9 cycles/deg: p = 0.001; 12 cycles/deg: p = 0.001; see Fig. 1).

Regarding the comparison of the results of contrast sensitivity for 6, 9 and 12 cycles/deg, the Friedman test showed a statistically significant difference in contrast sensitivity depending on the frequency of the target (see Fig. 1) for keratoconus (χ^2^ = 60.27, p < 0.001), for keratoconus with 0.00 LogMar visual acuity (χ^2^ = 32.70, p < 0.001) and for normal controls (χ^2^ = 70.00, p < 0.001). Post hoc analysis with Wilcoxon signed-rank tests with Bonferroni correction showed that better results were obtained for the lowest spatial frequency tested (for keratoconus, keratoconus with 0.00 LogMar visual acuity and for normal controls p < 0.001 for 6 vs 9, 6 vs 12 and for 9 vs 12 cycles/deg).

To see if the lower visual quality (e.g. contrast sensitivity) was a function of HOA, the correlation was between all ocular HOA and contrast sensitivity for 6, 9 and 12 cycles/deg was investigated (See Table 3). In the keratoconus group, most ocular HOA were negatively correlated with contrast sensitivity for 6, 9 and 12 cycles/deg (except tetrafoil and HOA spherical aberration for 6 cycles/deg). When the correlation was analyzed only in keratoconus subjects with visual acuity of 0.00 LogMar, all ocular HOA, except for HOA spherical aberration, were negatively correlated with contrast sensitivity for both 9 and 12 cycles/deg.

**Table 3:** Correlation between ocular higher-order aberrations and contrast sensitivity in 6, 9 and 12 cycles/deg for keratoconus group and keratoconus group with visual acuity of 0.00 LogMar.

Contrast sensitivity	Keratoconus (N = 38)			Keratoconus with VA 0.00 LogMar (N = 20)		
	6 cycles/deg	9 cycles/deg	12 cycles/deg	6 cycles/deg	9 cycles/deg	12 cycles/deg
Total RMS HOA	r = − 0.42** p = 0.009	r = − 0.56** p < 0.001	r = − 0.61** p < 0.001	r = − 0.30 p = 0.195	r = − 0.54* p = 0.014	r = − 0.58** p = 0.007
Trefoil	r = − 0.38* p = 0.020	r = − 0.47** p = 0.003	r = − 0.46** p = 0.009	r = − 0.33 p = 0.156	r = − 0.56* p = 0.010	r = − 0.52* p = 0.020
Total Coma	r = − 0.41* p = 0.011	r = − 0.55** p < 0.001	r = − 0.61** p < 0.001	r = − 0.31 p = 0.179	r = − 0.53* p = 0.016	r = − 0.57** p = 0.009
Tetrafoil	r = − 0.23 p = 0.172	r = − 0.36* p = 0.027	r = − 0.42** p = 0.009	r = − 0.40 p = 0.079	r = − 0.51* p = 0.022	r = − 0.62** p = 0.004
HOA astigmatism	r = − 0.44** p = 0.006	r = − 0.56** p < 0.001	r = − 0.65** p < 0.001	r = − 0.36 p = 0.125	r = − 0.62** p = 0.004	r = − 0.63** p = 0.003
HOA Spherical aberration	r = − 0.30 p = 0.071	r = − 0.41* p = 0.010	r = − 0.45** p < 0.001	r = − 0.08 p = 0.724	r = − 0.27 p = 0.251	r = − 0.29 p = 0.209

*Significant level of < 0.05, **significant level of < 0.005.

RMS, root mean square; HOA, higher-order aberrations; deg, degree; R, Spearman correlation results.

## Discussion

This study evaluated contrast sensitivity and ocular HOA in keratoconus subjects and keratoconus suspect subjects. Lower contrast sensitivity and larger ocular HOA were detected in keratoconus subjects compared to controls. However, more than half of the keratoconus cohort (20 subjects, 53%) had normal visual acuity of 0.00 LogMar, having also worse contrast sensitivity and more ocular HOA compared to controls. To elucidate the possible link between visual quality and ocular HOA, the relationship between contrast sensitivity and ocular HOA was specifically analyzed in keratoconus subjects with visual acuity of 0.00 LogMar. A negative correlation was found between most of ocular HOAs and contrast sensitivity in keratoconus subjects and keratoconus subjects with visual acuity of 0.00LogMar. For keratoconus subjects, almost all ocular HOA were negatively correlated with contrast sensitivity for 6,9 and 12 cycles/deg. For keratoconus subjects with visual acuity of 0.00 LogMar, a negative correlation was found between all ocular HOA (except tor spherical HOA) and contrast sensitivity for 9 and 12 cycles/deg. These findings suggest that HOA underlie the poor visual quality experienced by keratoconus patients with normal visual acuity.

Many studies evaluated HOA^20–26^ and contrast sensitivity^13,14,17,32,33^ on keratoconus patients. Just three previous studies^20,27,28^, analyzed both HOA and contrast sensitivity and the relationship between them in keratoconus subjects. However, none of them examined this relationship for keratoconus subjects with good visual acuity. The current study is the first to assess correlation between wavefront aberrometry measured by a Hartmann-Shack aberrometer and contrast sensitivity in patients with keratoconus and a subgroup of keratoconus patients who have good visual acuity (0.00 LogMar). Table 4 compares all the studies on the topic. Only one study measured ocular wavefront using a Hartmann-Shack aberrometer, as in the current study, but the patients had pupil dilation, which does not representation real-life vision^27^. The other two studies were focused on the analysis of HOAs calculated from the curvature (and elevation) of the anterior corneal surface^20^ or both surfaces^28^. In addition, one study^20^ tested the correlation between HOA and contrast sensitivity in 91 eyes with a variety of corneal conditions, including 8 eyes with keratoconus, but with no specific correlation only in eyes with keratoconus. Despite the differences in methodology, all found a similar negative correlation between HOA and contrast sensitivity.

**Table 4:** Description of studies that evaluated contrast sensitivity and higher-order aberrations in keratoconus subjects.

	Applegate et al. 2000^20^	Okamoto et al. 2008^27^	Bilen et al. 2016^28^	Current study
Control (N)	13 (13P)	26 (13P)	No	35 (35P)
KC (N)	8 (8P)	22 (14P)	71 (71P)	38 (22P)
KC (severity)	No data	No data	Early to moderate	Subclinical and Mild (grade 1–2)
Mean age of KC subjects (years)	No data	30.5 ± 8.4 No data	28.3 ± 8.3 14–54	25.6 ± 5.0 17–38
LogMar VA of KC group (range)	No data	0.04 ± 0.17 0.30 to − 0.10	0.25 ± 0.21 1.00 to 0.00	0.08 ± 0.12 0.50 to 0.00
Separate analysis for KC group with 0.00 LogMar BCVA	No	No	No	Yes
Pupil dilation	Yes	Yes	No	No
Pupil size (mm)	3 and 7	6	6	5
Aberrometry method	Calculated	Measured	Calculated	Measured
CS target	Horizontal sinusoidal bars	Letter	Letter	Vertical Gabor patches
CS method	Two alternative forced choice	CSV-1000LV chart	Hamilton-Veale chart	Two alternative forced choice
Main findings for KC: HOA and CS	Negative correlation between corneal aberration and CS	Negative correlation between CS and Third and Forth order aberration	Negative correlation between CS and total RMS and vertical coma HOA	Negative correlated between CS and HOA in KC group with 0.00 LogMar VA

N, numbers of eyes; P, numbers of participants; KC, keratoconus; CS, contrast sensitivity; RMS, root mean square; HOA, higher-order aberrations; deg, degree; VA, visual acuity.

The results of the current study confirm the relationship between the visual impairment in keratoconus subjects, even with good visual acuity, and the presence of higher amounts of HOAs compared to controls. However, the correlations between contrast sensitivity and ocular HOAs were moderate, suggesting the presence of other factors contributing to the limitation of the visual quality in keratoconus. Ocular scattering has been also found to be a limiting factor of the visual quality in keratoconus^17^. Jinabhai et al. found that intraocular light scatter was significantly greater in the keratoconic patients than in normal, with high levels of negative correlation between the magnitudes of intraocular straylight and baseline contrast sensitivity measured with the Pelli-Robson test in keratoconus eyes. More research should be conducted in the future to understand the exact contribution of HOAs and intraocular scattering to the degradation of contrast sensitivity.

Contrast sensitivity improvement in the human eye has been shown after correction of HOAs with an adaptive optics system^34^. Thus, it is possible that the decline in quality of daily life that has been previously reported in keratoconus subjects^6,7^ is directly related to the increase in HOA leading to a reduction in contrast sensitivity. In addition, the absence of visual acuity impairment in some cases in spite of high amounts of HOAs may be due to poor retinal image adaptation^35^ or cortical factors that limit visual acuity^36^. Indeed, visual acuity that was measured after using an adaptive optics system to correct aberration was worse in keratoconus eyes compared to healthy eyes, although both had similar retinal image quality^35^. Sabesan et al.^35^ argued that this may happen because keratoconus subjects experienced poor retinal image for a long period, and Rossi et al. (2007) claimed for cortical factors that limit potential visual acuity improvements^36^. Visual experience for a long term with a blurred retinal image might cause the visual system to become less sensitive to reduced image quality, as was reported for myopic blur^37,38^ and as was suggested recently for moderate keratoconus eyes^39^. Moreover, the contrast sensitivity test used in this study showed a "floor" effect at low contrast (due to the graphical limitations of the computer monitor) which may have affected the level of correlation among contrast sensitivity and HOAs. A more sensitive tool may have provided better resolution and lead to a stronger correlation between these two variables.

The current study provides important insight into contrast sensitivity in keratoconus patients because we used Gabor patches and an interval 2AFC staircase procedure. The Gabor patch, which is a sinusoidal gratings with a Gaussian envelope^40^ can be modulating for contrast and frequencies, and is considered the preferred method for testing contrast threshold since it can cause selective cortical responses for contrast and frequencies^41^. Furthermore, it gives good indication of real-world visual stimuli^42,43^. In contrast, the previous studies that tested the correlation of contrast sensitivity and HOA in keratoconus subjects, used Letter targets with constant size (i.e., no variety of frequencies)^27,28^. Only one study used horizontal sinusoidal bars^20^ using six spatial frequencies.

In the current study we tested contrast sensitivity with medium and high frequencies, that was shown to be reduced in keratoconus subjects before visual acuity decreases^19^. The adaptive staircase method with forced-choice procedure reduces the guessing rate^12^ and thus enabling more accurate threshold measurement. These differences in methodology used in the current study may provide a sensitive tool to assess the contrast sensitivity in keratoconus subjects with good visual acuity. This might explain the variance in sensitivity measurements among keratoconus subjects with similar visual acuity in our study.

In agreement with previous research^14,17^, our results show that contrast sensitivity was significantly lower for keratoconus subjects compared with healthy controls for the three spatial frequencies evaluated. Furthermore, contrast sensitivity was even significantly lower in keratoconus and keratoconus suspect subjects who had normal visual acuity compared to healthy controls for all spatial frequencies. In agreement also with other studies^13,32^ that used grating stimuli, contrast sensitivity was found to decrease with increasing frequencies. In addition, a higher contrast threshold was found for keratoconus subjects and keratoconus subjects with normal visual acuity compared to controls.

This study has some limitations that should be acknowledged. First, the cohort is limited, but it provides enough statistical power to extract valid conclusions about differences in contrast sensitivity among keratoconus and controls. A larger sample size would perhaps have been beneficial to yield in a more accurate display of the difference between correlation of contrast sensitivity and ocular HOA in keratoconus vs keratoconus suspect subjects. Second, there are age differences between groups, although not statistically significant, with keratoconus subjects being slightly older. Keratoconus is a progressive disease and therefore older subjects may show progressive signs and symptoms of keratoconus^1,44^, which may have an effect on contrast sensitivity^45^ and HOA^46,47^. However, the differences in ages are minor and since all our cohort had mild keratoconus and more than 26% of them were diagnosed as keratoconus suspect, the influence of this in the results may be marginal. Third, the measurements of ocular HOAs by the L80 wave + were calculated for only one pupil size (5 mm) so that comparisons with other studies that used different pupil size are difficult. In addition, the aberrometer in this study has yet to be validated, although this is a previous version of an aberrometric system validated afterwards^48–50^. Fourth, subjects removed their contact lenses only 30 min / a night prior the examination, depending on the type of lenses they wore (soft or hard lenses respectively), which may have influenced the results of the study. However, only 4 subjects wore contact lenses (3 of them with soft lenses) of which, only one had visual acuity for 0.00 LogMar. Therefore, we may assume that the results of the current study, especially of subjects with visual acuity 0.00 LogMar, were not affected by the wearing of contact lenses.

In conclusion, keratoconus subjects even with visual acuity of 0.00 LogMar have lower contrast sensitivity and higher ocular HOA compared to controls. A correlation between ocular HOA and contrast sensitivity in keratoconus subjects and keratoconus subjects with visual acuity of 0.00 LogMar confirms that the mechanism underlying the decreased vision quality in subjects with keratoconus and/or keratoconus suspect may be partially due to increased ocular HOAs.

## Material and methods

### Subjects

This study was approved by the Hadassah Academic College Ethics Committee and followed the tenets of the Declaration of Helsinki. Healthy, keratoconus and keratoconus suspect subjects (males and females) between the ages of 17–40 years participated in this study. Subjects above 40 were not recruited to the study since contrast sensitivity has been shown to be reduced above this age^51^. Subjects were recruited from the clinics and student body of Hadassah Academic College.

All examinations took place at the Hadassah Academic College eye clinic. The methods were orally explained to the participants and they signed a statement of informed consent prior to their participation.

Subjects were classified into two groups, keratoconus, and control groups. The keratoconus group included both keratoconus and keratoconus suspect subjects. Diagnosis of keratoconus was based on abnormal topography or tomography and at least one of the following signs^2^: stromal thinning, Munson’s sign, Fleischer’s ring, or Vogt’s striae, observed by slit-lamp examination or scissor reflex observed by a retinoscope. Keratoconus severity was graded using Amsler-Krumeich classification^3^. Only subjects with mild keratoconus (grades 1 and 2) were included in this study. The criteria for keratoconus suspect was abnormal topography and or tomography, but without clinical signs^52,53^. This group included eyes with early or forme fruste keratoconus (i.e., eyes of patients with clinically evident keratoconus in the fellow eye) and keratoconus suspects (i.e., corneas with tomographic signs of keratoconus but without evidence of clinical keratoconus in either eye), as defined by Klyce^53,54^.

Since keratoconus is an asymmetrical disease^2^, both eyes were tested and diagnosed^55^. For healthy controls, only one eye per patient (randomly assigned) was included in the analysis.

Subjects were excluded if they had any systemic or ocular condition positively or negatively associated with keratoconus^56^, including eye surgery such as corneal collagen cross-linking. Contrast sensitivity measurements were performed while subjects wore their best correction and not with contact lens since it has been shown to decrease scatter^57^. Subjects who wore soft contact lens were asked to remove those 30 min before the exam. For subjects who wore hard contact lens, wear was stopped the night prior to the exam^23,52^. Subjects with severe contact lens side effects (such as corneal warpage, scars) or who did not have full contrast sensitivity measurements were excluded from the analysis.

### Procedures

Subjects underwent a complete ocular exam. Monocular visual acuity was tested as a baseline measure using a modified Bailey–Lovie (LogMAR) chart (ETDRS) at 6 m distance with spectacle correction. Over correction was performed to determine best corrected visual acuity, and only subjects with maximum over correction of ± 0.50 DS continued with the protocol, with the modified prescription in a trial frame.

Autorefraction and total ocular wavefront aberrometry were measured with the L-80 wave + system (Visionix Luneau, Chartres, France), using the Hartmann-Shack method that measured HOA as opposed to instruments that calculate HOA^23,58,59^. The wavefront aberrations were described using Zernike polynomials, which are a set of complete orthogonal polynomials defined on a unit circle. A detailed description of analysis of the Zernike polynomials can be found elsewhere^21,23,60,61^. The ocular HOA were quantified using the root mean square as an index of the image quality. The lower the RMS value, the less aberrated was the optical system. All data from the wavefront analyzer database of the L80 wave + were extracted automatically by the instrument using a prototype program for Zernike vector analysis. The 35 Zernike polynomials for ocular HOA were calculated by the software of the L80 wave + ^62^.

Root Mean Square values were obtained by calculating the square root of the sum of the squares of j6 to j35, using the standard nomenclature for describing Zernike terms found in Atchison et al.^61^. From the 35 Zernike coefficients measured by the instrument, the following RMS groups were examined^60^: total RMS HOA (all terms included in the third, fourth, fifth, and sixth order), total trefoil (including j6, j9, j16, and j19), total coma (including j7, j8, j17, and j18), total tetrafoil (including j10, j14, j22, and j26), high order astigmatism (including j11, j13, j23, and j25), and total HOA spherical aberration (including j12 and j24). The Zernike polynomials were calculated for each subject using a 5-mm pupil.

Autokeratometry, corneal topography and tomography was performed using the Sirius system (Costruzioni Strumenti Oftalmici, CSO, Firenze, Italy). Slit lamp biomicroscopy and retinoscopy were performed to evaluate clinical signs of keratoconus. Each exam was performed by a licensed optometrist and the diagnosis confirmed by an ophthalmologist with a specialty in cornea.

Contrast sensitivity was tested with presenting spectacle correction (or with over-refraction if needed) using a previously developed psychophysical methodology^29,63–65^. Stimuli was presented on a Philips color monitor, using a PC (1024 × 768 pixels at a 100 Hz refresh rate; gamma correction was applied). Lighting conditions were calibrated with a photometer. The stimuli were presented at a viewing distance of 150 cm only to the tested eye using a diffuser for the fellow eye. Contrast sensitivity threshold was measured using a two-alternative forced-choice (2AFC) method^29,66^. Gabor patches (GPs) including vertically oriented sinusoidal gray-level gratings targets were presented in three blocks for 6, 9 and 12 cycles/deg consisting of 50 trials each. Each trial consisted of two stimuli presentation with a pair of images in which only one image randomly contained the target. Four peripheral high-contrast crosses appeared in each corner of the screen to inform the subject on target appearance. Before each trial, a small fixation circle was located to ensure central fixation before initiating the trial sequence. Each presentation was performed for duration of 100 ms (i.e., a transient/interval presentation) separated by an interval of 500 ms. A response was required in each trial using the computer mouse. A training session preceded the main experiment to ensure that subjects were familiar with the procedure. Thresholds was measured utilizing a 3:1 up-down staircase approach, which estimated the stimulus strength at a 79% accuracy level^67^. In this method, the target contrast was increased by 0.1 log units (26%) after an erroneous response and was decreased by the same amount after three consecutive correct responses. The subjects activated the presentation of each pair of images (i.e., a single trial) at their own pace. Auditory feedback was provided. Subjects with binocular keratoconus / keratoconus suspect or with one eye with keratoconus and the other with keratoconus suspect were tested in the psychophysical experiments twice in a random order.

### Statistical analysis

Statistical analysis was performed using SPSS version 25 (IBM Corp., Armonk, NY, USA). Normality was checked on each parameter for each group separately by means of the Anderson–Darling test. Fisher’s exact test was done to test gender differences between groups. Mann–Whitney test was performed to assess the differences between groups of subjects. Spearman correlation was performed to test the correlation between ocular HOA and contrast sensitivity. Friedman test and Post hoc analysis with Wilcoxon signed-rank tests were performed to compare contrast sensitivity in different frequencies (6, 9 and 12 cycles/deg) for each group of subjects. A p value < 0.05 was considered significant.

The statistical power associated to this sample was calculated a posteriori using the online calculator GRANMO (https://www.imim.es/ofertadeserveis/software-public/granmo/). Considering an unpaired comparison of two independent groups (control vs. keratoconus), the difference in contrast sensitivity for 12 cycles/deg between groups (1.53 log units), the sample size of each group (35 vs. 38), an alpha error of 0.05 and the standard deviation of each sample, the statistical power was found to be 99%.

### Ethical approval

All procedures performed in studies involving human participants were in accordance with the ethical standards of the institutional and/or national research committee (Hadassah Academic College Ethics Committee) and with the 1964 Helsinki declaration and its later amendments or comparable ethical standards.

### Informed consent

Informed consent was obtained from all individual participants included in the study.
